# The role of material deprivations in determining ART adherence: Evidence from a conjoint analysis among HIV-positive adults in Uganda

**DOI:** 10.1371/journal.pgph.0000374

**Published:** 2022-08-17

**Authors:** Uzaib Saya, Zachary Wagner, Barbara Mukasa, Peter Wabukala, Lillian Lunkuse, Sebastian Linnemayr

**Affiliations:** 1 Pardee RAND Graduate School, Santa Monica, California, United States of America; 2 RAND Corporation, Santa Monica, California, United States of America; 3 Mildmay Uganda, Mildmay Hospital and Institute of Health Sciences, Kampala, Uganda; PLOS: Public Library of Science, UNITED STATES

## Abstract

Despite sustained global scale-up of antiretroviral therapy (ART), adherence to ART remains low. Less than half of those in HIV care in Uganda achieve 85% adherence to their ART medication required for clinically meaningful viral suppression, leaving them at higher risk of transmission. Key barriers to ART adherence include poverty-related structural barriers that are inter-connected and occur simultaneously, making it challenging to examine and disentangle them empirically and in turn design effective interventions. Many people living with HIV (PLWH) make tradeoffs between these various barriers (e.g., between expenses for food or transportation) and these can influence long-term health behavior such as adherence to ART. To be able to estimate the distinct influence of key structural barriers related to poverty, we administered a conjoint analysis (CA) to 320 HIV-positive adults currently taking ART at an urban clinic in Uganda between July 2019 and September 2020. We varied the levels of four poverty-related attributes (food security, sleep deprivation, monthly income, and physical pain) that occur simultaneously and asked respondents how they would adhere to their medication under different combinations of attribute levels. This allows us to disentangle the effect of each attribute from one another and to assess their relative importance. We used regression analysis to estimate the effects of each attribute level and found that food security impacts expected adherence the most (treatment effect = 1.3; 95% CI 1.11–1.49, p<0.001), followed by income (treatment effect = 0.99; 95% CI 0.88–1.10, p<0.001. Sleep and pain also impact adherence, although by a smaller magnitude. Sub-group analyses conducted via regression analysis examine heterogeneity in results and suggest that the effects of material deprivations on expected adherence are greater among those with high levels of existing food insecurity. Results from this CA indicate that external factors inherent in the lives of the poor and unrelated to direct ART access can be important barriers to ART adherence. This study applies a CA (typically administered in marketing applications) among PLWH to better understand individual-level perceptions relating to poverty that often occur simultaneously. Policy interventions should address food insecurity and income to improve adherence among HIV-positive adults.

## Introduction

High levels of HIV incidence remain a major global public health concern. The Joint United Nations Program on HIV/AIDS (UNAIDS) estimates that there are 38 million people living with HIV (PLWH) globally, of whom more than half live in sub-Saharan Africa (SSA) [1]. Of the global population of PLWH, almost 27 million are now accessing lifesaving antiretroviral therapy (ART), a dramatic increase from the 7.7 million accessing ART in 2010 [1]. However, the benefits from treatment such as decreased HIV transmission, and improved health, quality of life, and life expectancy [2–4] depend on continued retention in care and continuously high medication adherence. Lack of adherence to therapy and sustained non-adherence over time could increase a person’s viral load and the risk for transmitting HIV. Current evidence suggests that the adherence rate required to achieve viral suppression is approximately 80% [5], and clinical studies from adult populations in SSA indicate that between 21–44% of adults taking ART have insufficient adherence to reap the full treatment benefits [6–8]. In Uganda, the location of this study, less than half of PLWH achieve 85% adherence to their ART medication, leaving them at risk of transmitting infection, disease progression, and HIV-related mortality [9].

Continued adherence to ART is largely determined by individual behavior and linked to broader structural issues [10, 11]. However, it remains unclear which barriers to adherence are the most important, making the design and targeting of interventions challenging. Structural adherence barriers include access to medication, stigma and discrimination, gender norms, poverty, competing priorities and unpredictability in schedule; individual motivational factors include self-efficacy and acceptance of HIV that require adapting ART into daily life [12]. In sub-Saharan Africa, where a majority of people with HIV live, barriers related to poverty and poor living conditions have been shown to influence individual-level decision-making. Material deprivations correlated with poverty can act as a “load” on cognitive function and decision-making [13], with effects that can be immediate (e.g. via physical pain) to slow-moving or cumulative (e.g., lack of sleep or appropriate nutrition). They can exacerbate behavioral biases, which can influence ART adherence [14]. Lack of sleep and presence of physical pain may not be easily detectable and can impair cognitive function (through its effects on attention and learning) and reduce memory and reasoning. There is a dearth of evidence on the role of such factors on well-being and health-related behaviors especially in settings such as in SSA [15]. These poverty-related barriers may occur concurrently and include lack of money, food insecurity, physical pain, and sleep [13, 15].

Evidence from the HIV literature indicates that factors such as lack of money and food insecurity are often related to disengagement from HIV care [16, 17]. Due to competing demands, individuals may make trade-offs between getting sufficient food to take with their medication (to avoid side effects of taking some of their medication on an empty stomach) and taking their treatment on time, leading to problems such as missed refill appointments and subsequently lowered adherence [18]. This may result in poor retention in medical care, and subsequent ART non-adherence. Indirectly, too little food may influence mental function as well, making it difficult to sustain attention or resist temptations, which may in-turn affect HIV-related health [15]. However, much less research has been done to document the consequences of sleep deprivation and pain on decision-making and health and economic outcomes in these populations even though poor sleep quality and pain are associated with poverty (which can in turn drive HIV morbidity and mortality) [19, 20]. A recent study from India examines the impact of improving sleep conditions among the urban poor in Chennai who disproportionately suffer from severe sleep deprivation due to poverty-related factors such as environmental irritants. When evaluating the impact of different interventions to improve sleep quality, the authors demonstrate that improved sleep (in the form of naps) can alter decision-making (e.g., savings, increase attention to incentives, and reduce present bias) [21]. Evidence has shown that almost half of PLWH in some settings (such as in Ethiopia or Nigeria) report significant sleep disturbances, including difficulty falling asleep, waking up at night, and reduced sleep time [22–24]. Poor sleep may be a contributor to disease progression and further HIV-related morbidity primarily because of how it impairs the immune system and affects physical performance, cognitive function and emotion that could then influence medication adherence [25, 26]. Physical pain is also common among PLWH (with some estimates suggesting 54%-83% of PLWH globally suffer from chronic physical pain) [27]. Pain may interact with biological, psychological, and social processes that could compromise health-promoting behaviors such as adherence and retention in care. For example, stigma due to physical pain among PLWH has been shown to decrease adherence by reducing retention in care and substantive patient-provider relationships [28]. Both sleep and pain may influence HIV clinical outcomes through their combined effects on mental health as well as on cognition and impairments to memory and reasoning [15]. Despite the varying levels of evidence on barriers to ART adherence, it is challenging to determine which poverty-related barriers are most salient as they may be inter-related and act simultaneously. It is important to isolate the effect of each barrier to know exactly where non-clinical, poverty-related interventions should be targeted.

Our study is the first to our knowledge to estimate the relative importance on participants’ expected adherence of several of these poverty-related barriers through a conjoint analysis (CA) administered to HIV-positive adults at an HIV clinic in Kampala, Uganda. Conjoint analysis studies and discrete choice experiments (DCEs) have long been shown to produce reliable predictions of health-related behaviors [29]. Deploying a conjoint analysis study in this setting also supplements the existing use of choice experiments and conjoint analyses focused on ART, of which only 30% have taken place in SSA and to-date have largely emphasized the factors determining choice and convenience of treatment rather than those related to adherence [30]. Our study can help guide the design of effective policy interventions for improving adherence. The results from the CA are valuable since they isolate the effect of each attribute separately, while holding other attributes constant, and are thus one of the first few applications examining a set of inter-connected adherence barriers. Findings from this CA can help prioritize policy interventions that consider external poverty-related determinants of ART adherence outside the clinical environment. Such interventions can help enhance the effectiveness of HIV treatment programs by mitigating existing material deprivations.

## Methods

### Study setting and recruitment

Between July 2019 and September 2020, the CA was administered as part of the 12-month assessment to all currently active 320 HIV-positive adults who were enrolled in a randomized controlled trial (RCT) called “Behavioral Economics Incentives to Support HIV Treatment Adherence” (BEST) (clinicaltrials.gov: NCT03494777) [31]. The two-year trial is testing the efficacy of using small lottery incentives to support ART adherence for treatment-mature HIV clients. Participants were all active patients at Mildmay Uganda Hospital which has a longstanding research collaboration with local partners (as well as the BEST study team). Mildmay is a non-governmental organization in Kampala, Uganda that specializes in the provision of free comprehensive HIV/AIDS prevention, care, and treatment services through outpatient and inpatient care for over 15,000 patients. In terms of its service offerings and patient demographics, Mildmay is similar other HIV care facilities in the region (such as The AIDS Support Organization or TASO); as a result, their practices may be considered as representative of HIV care in the area at other similar facilities.

For the original RCT, clinical records and electronic databases were used to identify eligible patients who were then recruited based on specific inclusion and exclusion criteria. Participants were all 18 years of age or older, received ART for two or more years, and had demonstrated recent adherence problems at the time of recruitment within the past six months based on clinical records (via lack of viral suppression, attending adherence counseling, or being classified as Stage 3 or 4 per WHO guidelines). Individuals were excluded if they were not mentally fit to provide informed consent, spoke neither English nor Luganda (the local language), were participating in any other adherence-related study, or were inconsistently using the trial-issued Medication Event Monitoring System (MEMS) cap to monitor adherence. Once eligible clients were identified, the team used the dates of their next clinic appointment to recruit them into the study, and the CA module was administered at the participant’s 12-month mark in the study.

### Ethics statement

We obtained ethics approval from the RAND Corporation’s Human Subjects Protection Committee (#2016–0956), the Mildmay Uganda Research Ethics Committee Institutional Review Board (#02013–2018), and the Uganda National Council for Science and Technology (#2394). Participants were explained that CA scenarios were strictly hypothetical. Written consent to participate in the study was obtained from all participants prior to the start of data collection once the survey objectives and procedures were explained at study enrollment. To ensure confidentiality, we separated personal identification information from the response data, and respondents were only identified through their clinic ID. Data management and safeguarding details were provided to both the RAND and Ugandan IRB. We kept any de-identified data on secure, encrypted, password-protected portals and laptops. No electronic file linked the primary data identifiers to assigned names or addresses.

### Design

In this CA, the material deprivations were described by their characteristics, referred to as attributes, with each attribute assigned an attribute level. To define these attributes and their levels, we used a combination of literature review, existing qualitative evidence, and analysis of quantitative data obtained via a baseline survey of the study sample conducted a year prior to the CA.

Qualitative interviews conducted at the time of recruitment into the RCT indicated that many study participants cited food and income-related issues as key challenges for taking their ART on time and as prescribed by clinicians. Prior qualitative assessments have also demonstrated that such challenges exist in Uganda more broadly [32, 33]. Quantitative data obtained from the study sample 12 months prior to the CA provided benchmarks for specific levels of certain attributes. For example, at the time of study enrollment, the median monthly income among respondents was USh 100,000, and 43% of the sample claimed to have cut the size of meals or skipped meals in the prior week. Another 45% of the sample said they faced financial problems in the recent 6 months in terms of loss employment or income. This level of financial distress is known to result in broader challenges for ART adherence [34]. At baseline, almost 40% of the study sample reported trouble falling or staying asleep or sleeping too much—they reported this problem occurring either every day or more than half or on several days of the week in a two-week period.

The CA design has four attributes linked to these four material deprivations on income, food, sleep, and physical pain (Table 1). The lowest level of the attribute indicates the “worst” possible level of that attribute. For each attribute included in the conjoint analysis, there were three levels, except for “pain” for which there are only two levels. This design had a total possible 3x3x3x2 or 54 attribute level combinations. Literature suggests using a full-factorial design i.e. if there are A attributes and all have L levels, then the full factorial includes L^A^ number of models [35]. This recommendation guided the number of profiles or choice sets we used, but since it was too large to be used in practice, we used a fractional factorial design as other studies have done [36, 37]. We divided the fractional factorial design combinations in blocks, so that each participant was asked a maximum of eight questions (or “scenarios”).

**Table 1:** CA characteristics.

Attribute	Level 0 of attribute	Level 1 of attribute	Level 2 of attribute
Food insecurity	Skipped 2 or more meals	Skipped 1 meal	Skipped 0 meals
Pain	Physical pain that makes it difficult to perform daily activities	NA	No physical pain that makes it difficult to perform daily activities
Sleep	Less than 3 hours of sleep last night	3–5 hours of sleep with interruption	8 hours or more of sleep last night
Money	had no income in the last month.	Earned USh 100,000 in the last month.	Earned USh 200,000 in the last month.

In this study, we employed a CA with five levels of adherence (or choice options) where respondents are presented with eight different hypothetical scenarios or profiles within one block, randomly varying the levels of four attributes. They are then asked how they would adhere to their medication in each scenario. In this CA, the adherence levels (1–5) are the choice options [38, 39]. Deploying these choice options in this way within a CA module enables us to retrieve the preferential value or utility of each attribute level relative to others, so that the marginal utility derived from each attribute can be estimated independently. It is important to note that this CA module relies on the theory of conjoint measurement, which is not necessarily about the behavior of choices, but in fact about the individuals’ response to factorial manipulations of factor levels within attributes. The key assumption for our results to be meaningful is that the utility values we estimate from the CA are a good proxy for real adherence [40].

After each scenario, participants were asked about their likelihood of adherence using the self-rating scale item (SRSI), a Likert-based, self-report rating of ART adherence [41]. The SRSI has been significantly correlated with other self-report adherence items and inversely correlated with known predictors of medication nonadherence, such as illicit substance use and depression, indicating it’s suitability for measuring adherence. The SRSI had also predicted CD4 cell count and viral load as well or better than other adherence items, indicating good criterion validity [42]. Therefore the SRSI can be considered an effective but brief way to routinely measure self-report adherence in clinical settings.

Participants are randomly assigned to one of equally sized eight blocks. We generated 54 scenarios of the four attributes across these eight blocks. Because each block presented eight unique profiles, we did not need to exclude blocks where the level of one attribute dominated the other (i.e., at least as good on all attributes). Randomization ensured that this design was well-balanced, or that attribute levels within the randomized blocks appeared with comparable frequency and that the scenarios were balanced across the respondent sample [36].

The design was also orthogonal i.e., the different attribute levels varied independently and were uncorrelated with other attribute levels. We tested this by looking at pairwise correlations of the different attributes against one another; none of the correlation coefficients were significant or highly correlated. If attribute levels were correlated, it would be challenging to disentangle which attribute was driving preferences (for example if missing 0 meals was more likely to be paired with higher levels of income than missing 2 meals, the comparison of missing 0 and 2 meals would be confounded by the income effect).

### Data collection and procedures

**Fig 1:**
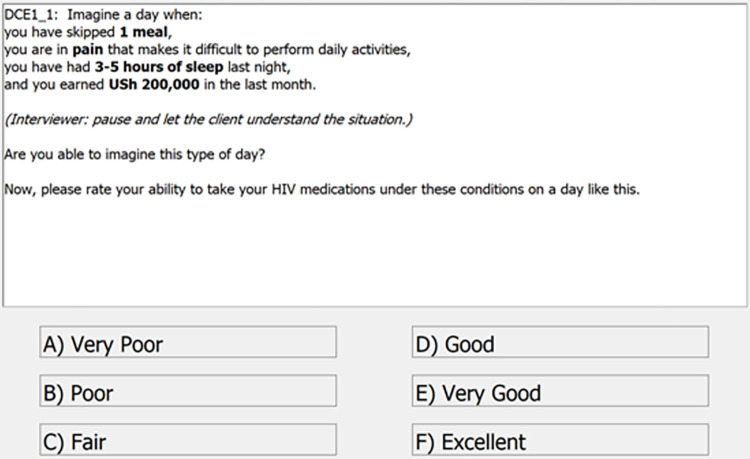
Screenshot of a scenario presenting different levels of the four attributes.

The CA was administered using electronic tablets, and the scenarios were read out by study staff in either English or Luganda, the local language (Fig 1). Before administering to study participants, study staff piloted the CA on a small number of non-participants to ensure language and content compatibility (e.g., to ensure specificity around the type of pain, study staff clarified that this was not meant to be life-threatening or emergent pain). The staff were trained to describe the hypothetical scenario as outlined and then asked the respondents if they were able to imagine this situation. All respondents were asked 3 practice scenarios before the start of the CA module, so that they could become familiar with the format. The practice scenarios helped respondents anticipate the types of questions especially in terms of imagining the daily situations associated with material deprivations. On average, the CA module in total took 10.6 minutes (SD: 4.0 minutes) per respondent. The Protocol for data collection is available in S1 File.

### Statistical methods

We estimate how expected adherence was impacted by each attribute level in accordance with standard conjoint analysis methods [43]. We calculated the average marginal component effect (AMCE) for each attribute level, which represents the average effect of that attribute level on expected adherence compared to a reference attribute level (the “worst” level within each attribute). To identify the effect of each attribute on the expected adherence, we estimated the following equation using ordinary least squares (OLS) regression. While OLS produces unbiased and consistent estimates of the difference in mean adherence between attribute levels [43] and is easy to interpret, other non-linear models are often more efficient. We conducted additional analyses using ordered logistic regressions as a robustness check, which could be more efficient but less straightforward to interpret.


$${Adherence}_{ic}=\lambda_{c}+\beta_{1}{\left(Skipped\;0\;meals\right)}_{ic}+\beta_{2}{\left(Skipped\;1\;meal\right)}_{ic}+\theta_{1}{\left(3-5\;hours\;of\;sleep\right)}_{ic}+\theta_{2}{\left(8\;hours\;of\;sleep\right)}_{ic}+\alpha_{1}{\left(Income\;of\;USh\;100,000\right)}_{ic}+\alpha_{2}{\left(Income\;of\;USh\;200,000\right)}_{ic}+\rho_{1}{\left(no\;pain\right)}_{ic}+\epsilon_{ic}$$

In this equation, *Adherence* is the Likert-response variable showing the probability of expected adherence in block *c* for person *i*. The *β*s represent the tradeoffs among the food-related attributes or the impact of switching from skipping 2 meals to skipping 0 or 1 meals on expected adherence, the *θ*s represent the impact of switching from less than 3 hours of sleep to 3–5 or 8 hours of sleep, the *α*s represent the impact of switching from no monthly income to 100,000 Ugandan Shillings (USh) or USh 200,000 of monthly income, and the *ρ*_1_ represents the impact of switching from pain to no pain that makes it difficult to perform daily activities. Leaving out the reference category in this regression avoids the dummy variable trap. To address any potential confounding introduced by chance imbalance due to the realities of randomization in practice, we include a set of block fixed effects (*λ_c_*) in our regression model, which holds constant any differences across blocks and estimates the impact of attribute levels within blocks. They adjust for fixed differences between blocks and improve precision. Standard errors are clustered at the individual level to account for within-respondent correlations between expected adherence (since each respondent contributes 4 observations) [44]. All analyses were done in Stata/MP 16.1 [45]. We assumed that the order of the choices presented in the block does not influence the outcome, that outcomes would remain stable across the blocks, and that the scenario presented to a respondent in one part of the block did not affect the response in the current task/choice [38].

We estimated the relative importance of each attribute by calculating the share of the variation explained by the full model (all attributes combined) that is attributable to each individual attribute. This involved estimating separate regressions for each attribute (excluding the other attributes) and dividing the R-squared from each separate model (the share of the variation explained by the respective attribute) by the R-squared of the full model (the share of the variation explained by all attributes). We estimated 95% confidence intervals of our importance estimates using a bootstrapping method. This involved resampling respondents with replacement, re-calculating the importance estimates, and repeating 1,000 times. We then used the empirical distribution from the 1,000 resamples to create 95% confidence intervals (2.5^th^ percentile is the lower bound and 97.5^th^ percentile is the upper bound).

We conducted subgroup analyses examining differences across various types of participants. These were done by running analyses separately for each subgroup (rather than interacting characteristics with attribute levels and then assessing the interaction terms in the main analysis). First, we assessed differences based on participants’ measured adherence levels. This was done via readings from the electronic MEMS caps and pill bottles given at the start of the study—these are used to record each time participants opened their ART pill container. We anticipate that those who are already adherent to their medication will be less sensitive to the material deprivations we are analyzing. Adherence was expressed as a percentage, calculated as the share of prescribed pills taken on each day (i.e., number of actual bottle openings divided by the prescribed bottle openings) during the three-month period preceding the CA. However, even MEMS data can be subject to measurement error, such as due to a novelty effect (e.g., when receiving the caps people may open it frequently out of curiosity, or they open the cap without taking the pill). In our study, participants had been using the MEMS on average for 10 months already, hence any measurement error due to novelty or learning effects would likely not be present at that stage. We are not able to distinguish between non-use of the MEMS cap and missed doses, which is a problem for most electronic adherence analyses. However, we excluded clients who were unable or unwilling to consistently use the MEMS cap from the study, so we do not expect non-use of the MEMS cap to be very common. Even with this limitation, MEMS cap measured adherence is still considered the gold standard for adherence measurement [46]. MEMS data were categorized as low adherence (up to 50th percentile, or adherent to 85% of doses), and high adherence (beyond 50^th^ percentile or above 85% adherence).

Secondly, we anticipate that expected adherence of those facing existing deprivations in food security will be more sensitive to changes in food security levels in the CA, because the missing meals are more salient for these people and because food insecurity is a significant barrier to ART adherence. Literature suggests various pathways by which existing food insecurity shapes ART adherence, namely increased hunger with ART; worsened side effects from ART without sufficient food; participants skipping doses if food was unavailable; competing demands for limited resources (especially between food or medical expenses) and forgetting to take ART while working for food [47]. We examined differences by whether participants were food insecure based on the Food Insecurity Experience Scale. We defined the levels as low (raw score 0–3) and high (raw score 4–5) based on whether the respondent responded affirmatively to questions on cutting the size of meals or skipping a meal, gone a whole day without eating, been hungry but couldn’t eat because they did not have money to buy food, not been sure where getting next meal, or felt worried or stressed about not having a reliable source of food. Thirdly, we examine differences in average monthly income levels (approximately US $37 in our sample)- those with lower reported income would be more likely to be responsive to changes in material deprivations because they are more likely to have experienced these deprivations. Lastly, we also explore differences by male and female respondents to examine demographic differences. All subgroup analyses were conducted using regression analyses. We followed the guidelines for Conjoint Analysis Applications in Health as outlined by the ISPOR Good Research Practices for Conjoint Analysis Task Force (S1 Checklist) [48].

## Results

Between July 2019 and September 2020, we administered the CA to 320 participants. Of these, 64% were female with an average age of 38 years (IQR: 27–47), 54.5% had some secondary education, the average monthly income was USD 37.30, and 20% reported high levels of food insecurity in the past week as measured by the Food Insecurity Experience Scale (Table 2) [49]. They had been on ART for almost 10 years on average and almost 87% of participants were virally suppressed. The full Mildmay Cohort in comparison was almost the same age with an equivalent number of female clients and had been on ART for relatively fewer years (7.3 years) and had a very slightly lower proportion who were virally suppressed (85%).

**Table 2:** Demographics and clinical characteristics of full Mildmay Cohort and CA Cohort as of September 2020.

Panel A		
**Characteristic**	**CA Cohort**	**Full Mildmay Cohort**
**N**	**320**	**15,777**
Age (years)	38.3	38.5
	(0.709)	(0.109)
Male (%)	36.5	35.6
	(2.69)	(0.381)
Years at Mildmay	11.4	7.93
	(0.214)	(0.042)
Years on ART	10.2	7.34
	(0.198)	(0.038)
Undetectable viral load (%)	87.1	85.4
	(1.86)	(0.281)
Panel B		
**Characteristic**	**CA Cohort**	
**N**	**320**	
English Preferred Language (%)	26.2	
	(2.45)	
Literate (%)	57.9	
	(2.76)	
Completed Secondary School (%)	54.6	
	(2.78)	
In Relationship (%)	53.7	
	(2.78)	
Monthly Income (USD)	37.3	
	(3.30)	
Employed (%)	62.1	
	(2.71)	
Time to Reach Clinic (Hours)	1.99	
	(0.074)	
Food Insecure (high %)	19.6	
	(2.22)	
Depressed (%)	6.87	
	(1.41)	
Impatient (%)	76.8	
	(2.35)	
Present Biased (%)	9.68	
	(1.65)	
Comfortable Disclosing Status (%)	74.3	
	(2.44)	
Adherence last month (scale %)	73.5	
	(0.015)	
Intrinsic Motivation Scale	6.68	
	(0.028)	

Notes Panel A: Data are from the electronic health records at Mildmay as of September 2020. Standard errors in parentheses. Viral load measures were based on recent viral load tests done prior to that; undetectable viral load refers to percentage of clients who are virally suppressed (defined as having less than 200 copies/mL).

Notes Panel B: Data are from the BEST study survey. Standard errors in parentheses. The electronic health data do not have relevant data for a variety of other data and/or measures obtained from the study survey which is why this panel excludes data on other participants not in the CA sample. Adherence was based on most recent MEMS reading prior to survey and expressed as a share of prescribed pills that were taken each day (calculated by dividing the number bottle openings on each day by the number of doses prescribed). Survey-based measures are noted below:

• Food Insecurity is based on responses to five survey questions on access to food at the level of the household and the associated constraints on ability to obtain adequate quantity of food (adapted from the Food Insecurity Experience Scale). We defined the levels as low (raw score 0–3), and high (raw score 4–5) based on whether the respondent responded affirmatively to questions on cutting the size of meals or skipping a meal, gone a whole day without eating, been hungry but couldn’t eat because they did not have money to buy food, not been sure where getting next meal, or felt worried or stressed about not having a reliable source of food.

• Depression was measured using the nine-item depression scale of the Patient Health Questionnaire (PHQ)-9, with each item scored on a Likert scale with symptoms rated as 0 (not at all), 1 (several days), 2 (more than half the days) and 3 (nearly every day). Those scoring more than 10 were categorized with major depression (or categorized as “Depressed”).

• Intrinsic Motivation Scale is a multidimensional self-report, Likert-type rating scale used to assess *motivational* structures for targeted activities (such as adherence) and scored using the Intrinsic Motivation Inventory which is the average across all the items in each of three sub-scales.

• For the behavioral economics questions, we categorized respondents as being present-biased if there was a discrepancy between preferences in waiting for same money at two different time periods. We defined respondents as being impatient if they did not want to wait for the larger sum of money.

• MEMS-measured adherence can suffer from measurement problems. We excluded participants who were unable or unwilling to use their MEMS caps. Like in other studies, we found that a negligible proportion of the sample pocketed doses to take them later in the day (especially if they were on a two-dose a day regimen) and so that they did not have to carry their pill bottle with them.

**Fig 2:**
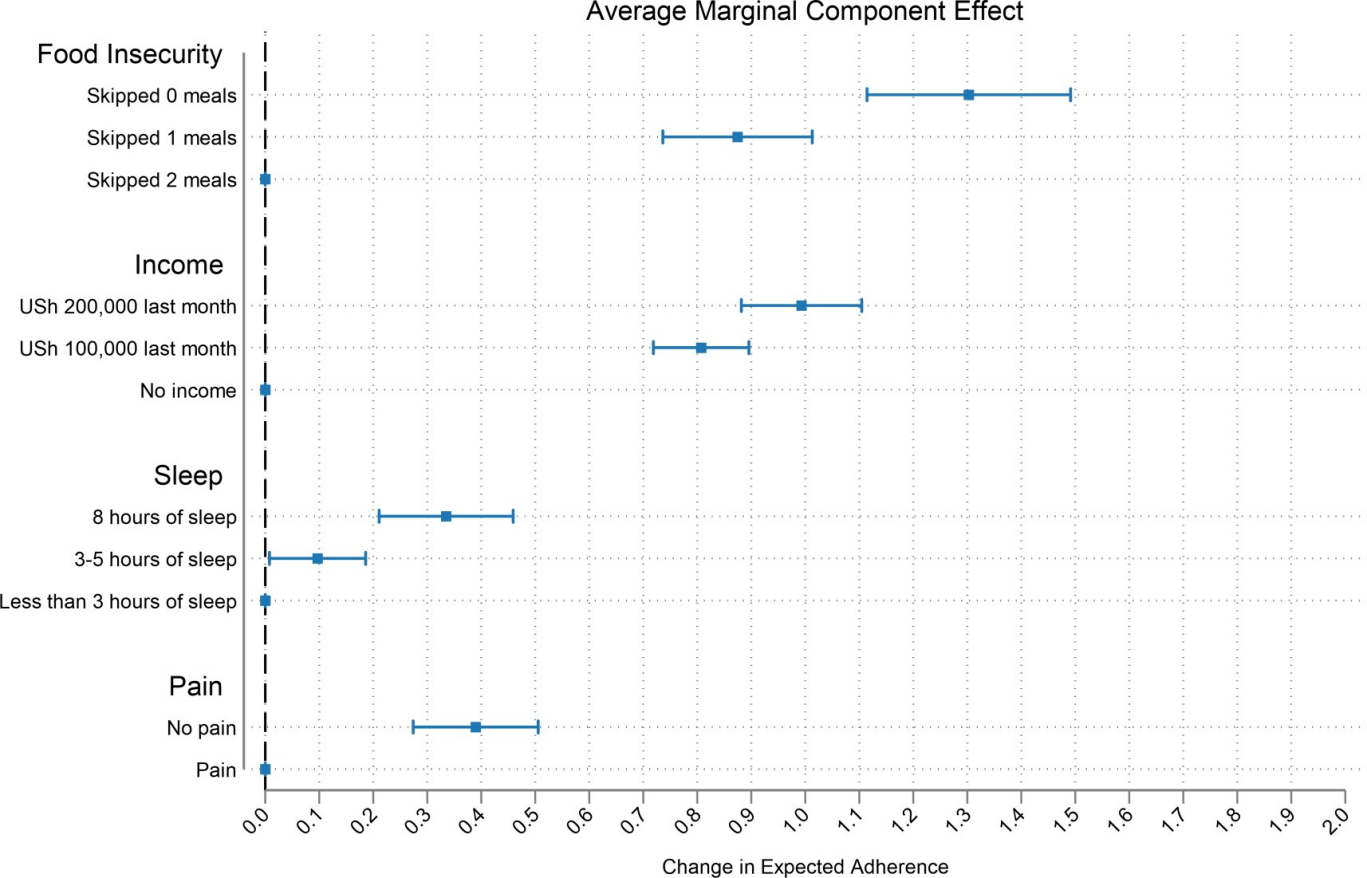
Average marginal component effects of material deprivation attributes on expected ART adherence. Note: Effect size estimates are based on regression estimators with standard errors clustered at the individual level, and horizontal bars represent 95% confidence intervals. The points without horizontal bars on the dashed vertical red line represent the attribute level that is the reference category for each attribute.

Changes in food security impacted expected adherence the most (treatment effect = 1.3; 95% CI 1.11–1.49, p<0.001), followed by improvements in monthly income (treatment effect = 0.99; 95% CI 0.88–1.10, p<0.001. Improvements in sleep and pain also increased adherence, although by a smaller magnitude (treatment effect for sleep = 0.34; 95% CI 0.21–0.46, p<0.05, treatment effect for pain = 0.39; 95% CI 0.27–0.51, p<0.001, Table 3 and Fig 2). The OLS coefficients can be interpreted as the change in the expected adherence when a given attribute value is compared to the baseline (or “worst” possible outcome in that attribute). Results from an ordered logistic regression show that the proportional odds ratios have the same direction and a similar magnitude as our OLS coefficients. Since attribute levels were randomly assigned, participant characteristics are uncorrelated with attribute levels in expectation. Thus, adding additional control variables does not affect results, as shown in S2 File.

**Table 3:** Main effects.

Attribute	Level	1			2		
		Coefficient	95% CI	p-value	Coefficient	95% CI	p-value
Food Insecurity	Skipped 0 meals	1.303	1.114–1.491	<0.001	6.692	5.437–8.236	<0.001
	Skipped 1 meal	0.875	0.736–1.013	<0.001	3.414	2.831–4.117	<0.001
Sleep	3–5 hours of sleep	0.0968	0.00774–0.186	<0.05	1.136	1.010–1.278	<0.05
	8 hours of sleep	0.335	0.211–0.459	<0.001	1.673	1.420–1.971	<0.001
Income	Earned income of Ush 100,000 in last month	0.807	0.719–0.896	<0.001	3.314	3.034–3.619	<0.001
	Earned income of Ush 200,000 in last month	0.993	0.882–1.105	<0.001	4.527	3.950–5.187	<0.001
Pain	No pain that makes it difficult to perform daily activities	0.390	0.274–0.505	<0.001	1.785	1.557–2.045	<0.001
R-squared		0.259					
Observations		2,560					
Number of participants		320					
Number of blocks		8					

Note: Column 1 shows results from the CA sample analyzed using an OLS regression specification, while column 2 shows results from the CA sample analyzed using an ordered logit specification. All coefficients represent improvements in expected ART adherence (coefficients in ordered logit column represent odds ratios) and are compared to the reference categories (Food Insecurity: Skipped 2 meals, Sleep: 0 hours of sleep; Income: No income; Pain: pain that makes it difficult to perform daily activities). 95% confidence intervals and p-values are shown adjacent to coefficients. Standard errors were clustered at the individual level.

We estimated the R-squared value as 26% in the full model (or the model sum of squares from the regression as a share of the total sum of squares). Food Insecurity alone explained 62.7% (bootstrapped 95% CI 54–71%) of the variation in expected adherence (or the reduction in adherence) that was explained by the full model, while income explained 27.2% (bootstrapped 95% CI 20–35%) of the variation in expected adherence (or the reduction in adherence) that was explained by the full model (Table 4).

**Table 4:** Assessing importance of each attribute (bootstrapped 95% CI).

Attribute	Explained Variation (Upper)	Explained Variation (Lower)
Food Insecurity	70.6%	54.3%
Sleep	1.7%	0.0%
Income	34.5%	20.0%
Pain	15.6%	5.2%

Note: The percentages are 95% CI that refer to the proportion of the total variation in self-reported adherence that is explained in the full model.

**Fig 3:**
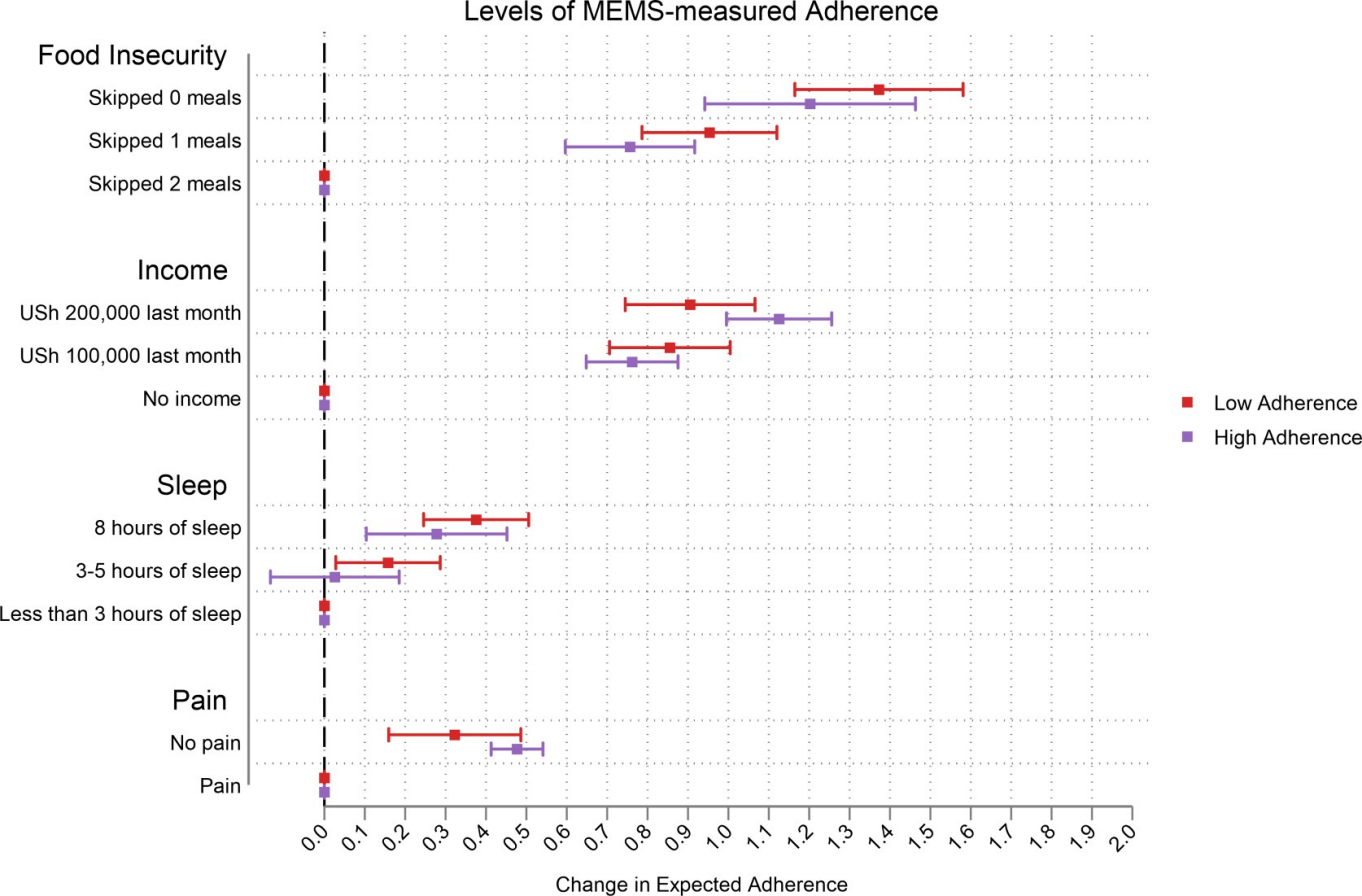
Effect of MEMS-measured adherence levels.

**Fig 4:**
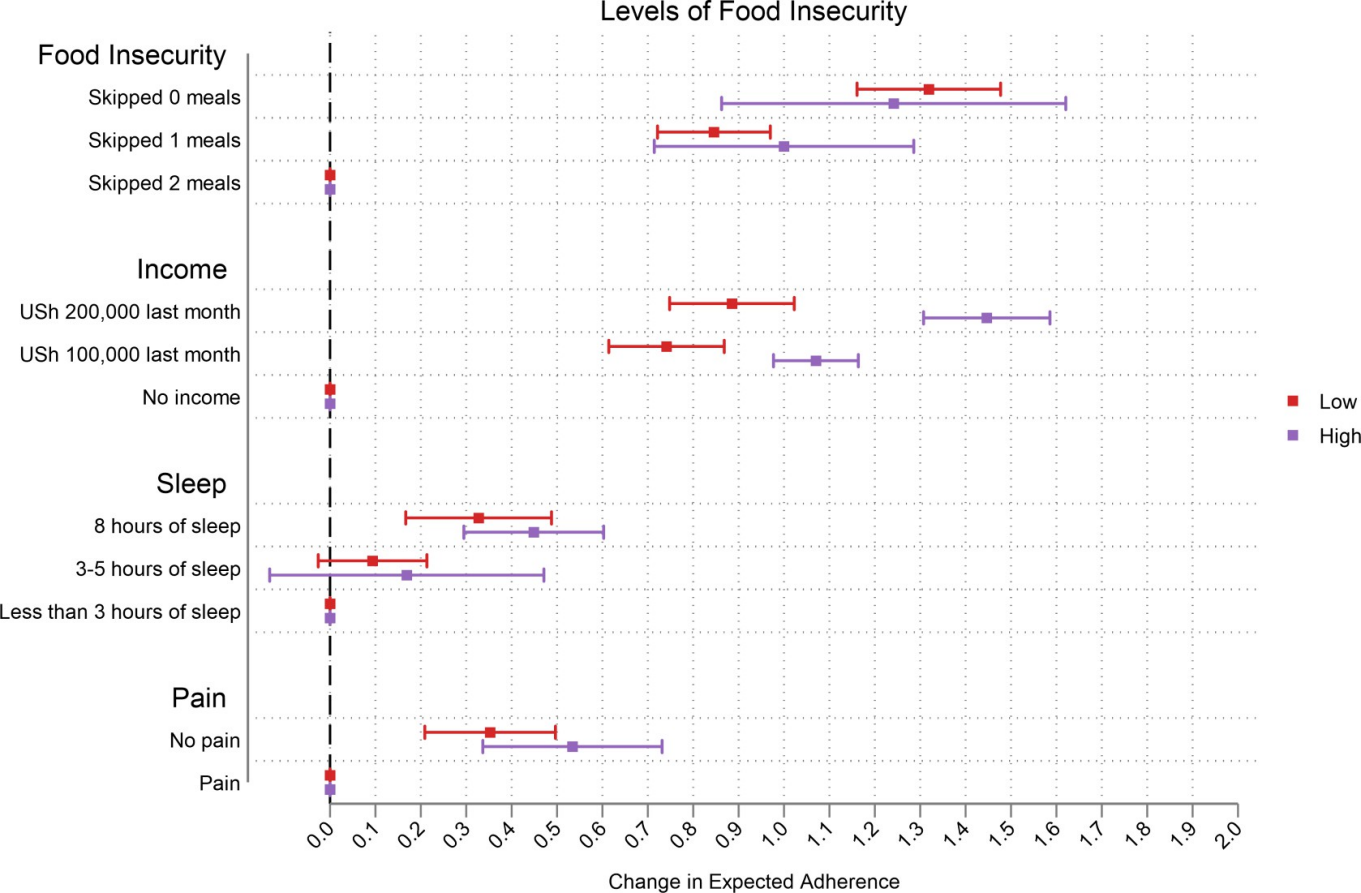
Effect of food insecurity levels.

**Fig 5:**
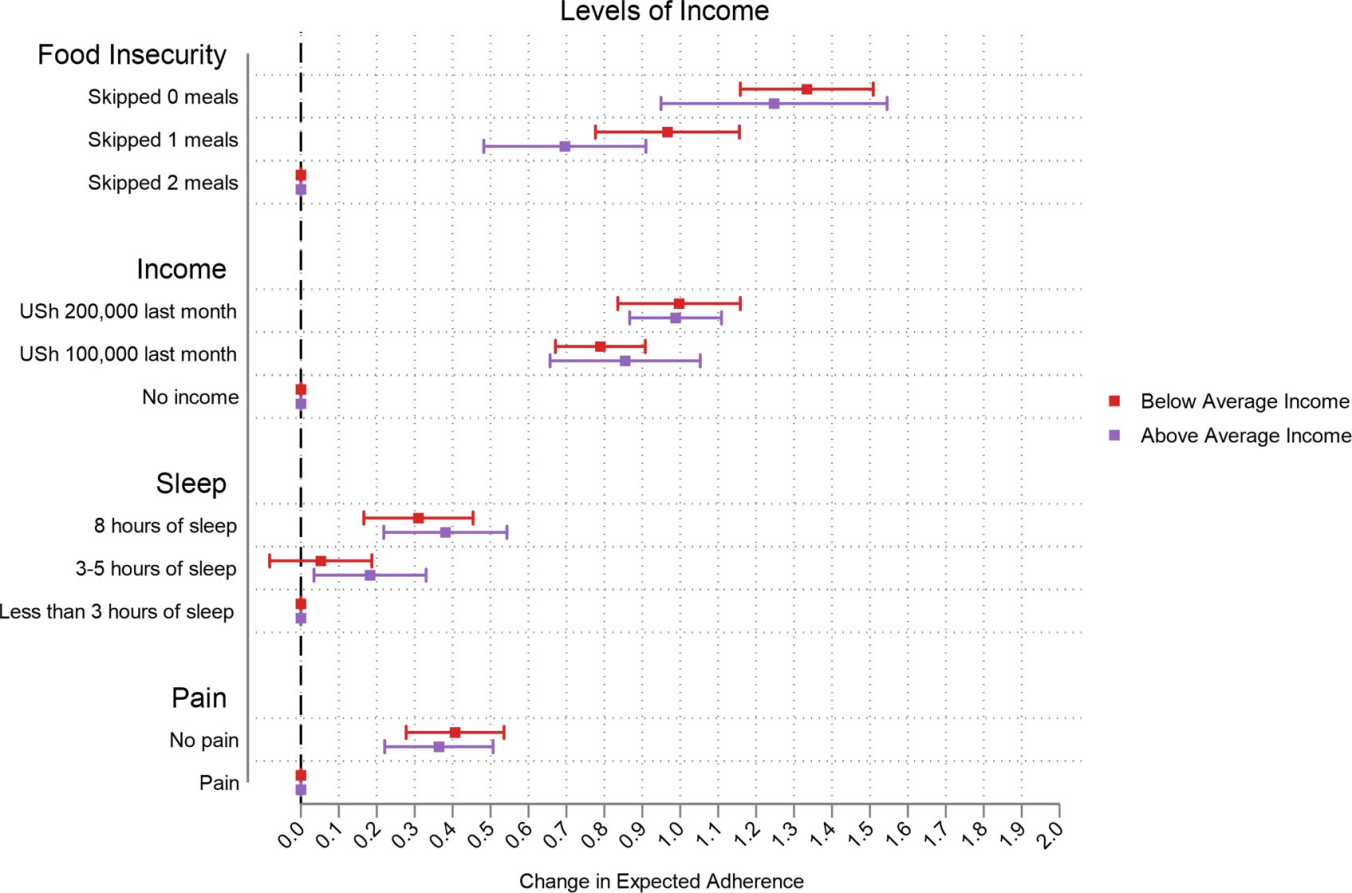
Effect of average monthly income levels.

**Fig 6:**
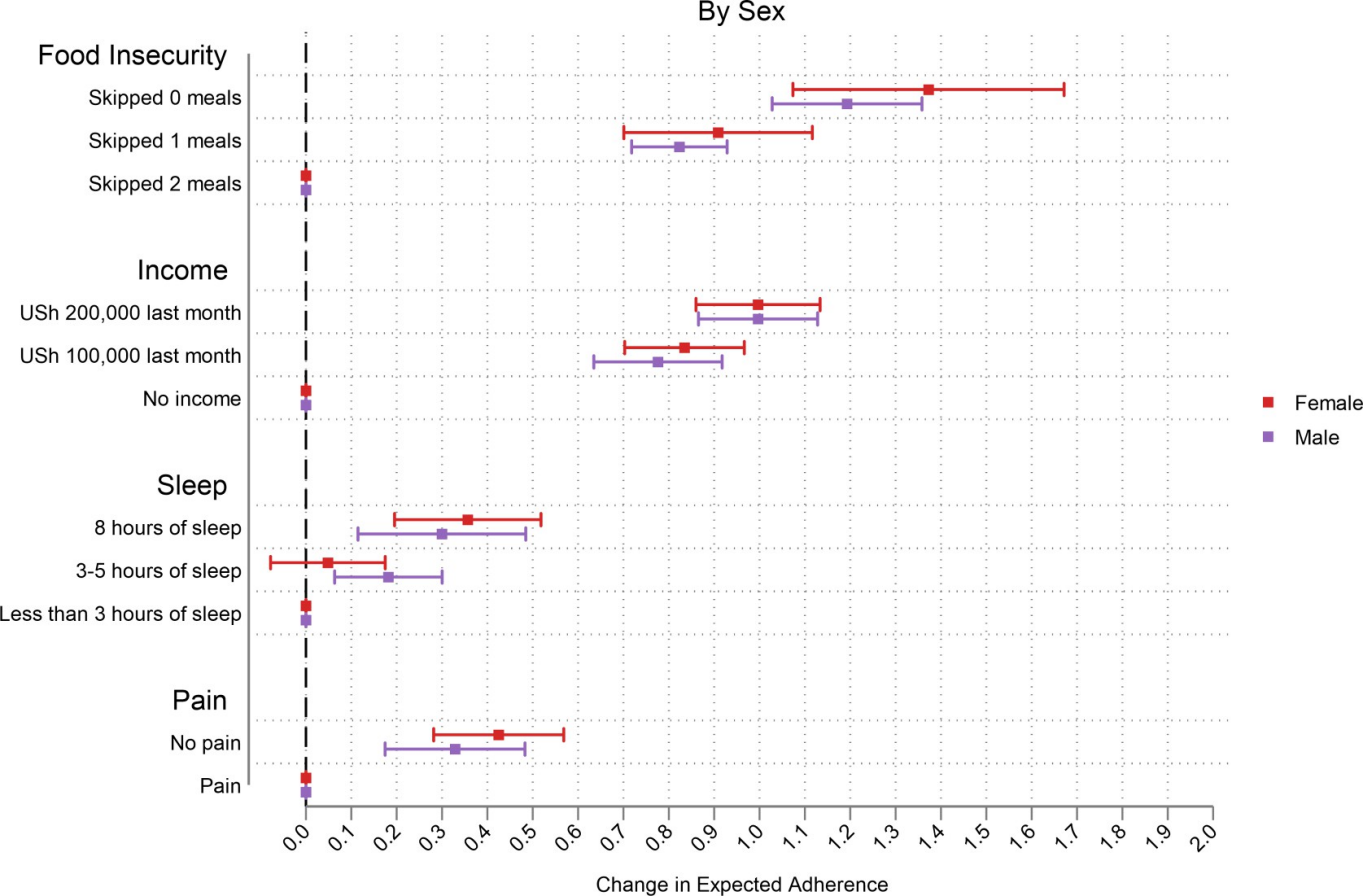
Effect of sex on adherence levels.

Those with low levels of MEMS-measured adherence reported greater changes in expected adherence, especially at higher levels of attributes (Fig 3). The change in expected adherence was much greater among those who were more food insecure especially in the income attribute. In this sample, switching to higher monthly income and food security in the CA increased expected adherence the most (Fig 4). We observed that the change in expected adherence was greater among those with below average income (and this was more prominent for the food insecurity attribute) (Fig 5). Changes in expected adherence were similar among male and female respondents but lower levels of food insecurity among female respondents resulted in higher expected adherence. Both sets of respondents reported higher change in expected adherence when switching to higher monthly income (Fig 6).

## Discussion

We conducted a CA among adults in HIV care in Uganda to provide insights into key material deprivations that influence expected medication adherence. We find that food insecurity and income-related factors play a greater role in determining changes in adherence relative to other measured deprivations such as lack of sleep or presence of physical pain. These results highlight the need for adherence interventions designed with such factors in mind.

Sub-group analyses serve to examine heterogeneity in results and suggest that the effects of material deprivations on expected adherence are greater among those with high levels of existing food insecurity, and those earning below average income. This indicates that those who often experience food insecurity are aware that it affects their adherence whereas people who do not miss meals might not even know that this would impact their adherence.

This study makes several contributions. First, our study results demonstrate that among well-known and inter-connected determinants of ART adherence, food insecurity and income play key roles. Our findings provide a sense of the relative contribution of each determinant, and this is a novel application of using a CA to understand how to disentangle important predictors of ART adherence in this population. In the context of HIV, food insecurity is an essential factor since medication is taken alongside food to avoid unpleasant side effects. Food insecurity can also heighten existing HIV-related vulnerabilities by disrupting daily routines, impairing memory and attention, and reducing motivation, resulting in lowered odds of achieving complete HIV viral suppression, poor ART adherence, and disengagement from care [17, 18, 50], Prior literature outlines the inter-connected relationship between food insecurity and ART, describing how taking ART regularly may help in alleviating food insecurity through greater ability to earn income, and at the same time, alleviating food insecurity could help improve ART adherence [51]. Our study’s findings indicate that food insecurity plays a greater role than income in determining adherence. Evidence suggests that PLWH make tradeoffs between expenses for food or transportation to the clinic, and fear of ART-related side effects in the absence of sufficient nutritional intake can deter adherence [18]. Food assistance in this context can act to improve economic well-being in the same way that cash transfers have been documented elsewhere as having an “income effect” [52] and can decrease barriers to care and adherence. Such assistance can also improve ART adherence by increasing motivation and changing the costs of other determinants of adherence such as transportation to the clinic. Our study also corroborates evidence from prior literature on the disproportionate burden of food insecurity challenges facing female HIV-positive adults: a previous meta-analysis found that the odds of developing food insecurity among female HIV-positive adults receiving ART is 53% more than male HIV-positive adults [53]. In our study, food security also has a larger effect on expected adherence among female respondents.

Additionally, while sleep and pain both have smaller effects on expected adherence in our study sample, worsening levels of these factors may be correlated with depressive symptoms among PLWH [54]. Increasing fatigue (and relatedly poor quality of sleep) and pain are somatic symptoms of depression which may be caused by HIV illness, and they can also make ART adherence worse [55]. Evidence from different settings in Ethiopia and South Africa shows increased odds of poor sleep quality among depressed PLWH [23, 56]. Smaller impacts of these factors on self-reported adherence may not be surprising given that only 7% of our study sample reported high levels of depressive symptoms (Table 3).

Secondly, our findings also demonstrate the need for future research examining how poverty shapes decision-making. Literature suggests that poverty may operate through multiple channels such as scarcity or stress. Poverty impedes cognitive functioning through scarcity, and it creates an increased focus on money resulting in lowered bandwidth for other tasks, while stress can in turn reduce productivity and income [57]. While research has established that factors such as material poverty (or lack of money) and food insecurity influence both physical and mental function, [15, 57] much less research has been done to document the consequences of sleep deprivation and pain on decision-making and health and economic outcomes. Sleep deprivation has been shown to reduce trust, influence risk attention and logical reasoning in US populations, and the presence of physical pain has led to individuals being more risk averse and making sub-optimal financial decisions [15]. Given that the decision to adhere to ART, and plan and execute long-term health in this way is inherently a cognitive process with rational and irrational decision-making processes, the findings from this study can help researchers and policymakers better understand the consequences of such external factors for long-term ART adherence among PLWH.

Another contribution of our study is that it examines hypothetical improvements in material deprivations that have important policy implications for decisionmakers interested in improving health and economic outcomes among PLWH outside the clinic setting. Evidence on existing policy interventions such as food assistance or other food-related interventions shows that these programs may affect the mechanisms through which food insecurity reduces adherence to ART. Food supplementation programs in Zambia, Malawi, and Haiti improved clinical outcomes including in some cases also adherence to ART (as measured by medication possession ratio, which is dependent on obtaining pharmacy refills) [18, 58]. Another study from Tanzania demonstrated that short-term cash and food incentives increased ART possession and retention in HIV care and can also in turn improve food insecurity [52, 59] A recent study in Uganda did not find any effects of unconditional cash grants on improvements in outcomes related to health and food security, but the authors did not use directly measured clinic outcomes such as viral load or electronically measured ART adherence [60]. Future policy interventions can enhance the effectiveness of HIV treatment programs by mitigating existing material deprivations. Recent literature has examined the effects of poverty and negative income shocks on higher levels of stress and possibly differential levels of economic decision-making [13]. Policy interventions and lines of future research can examine exactly how such factors impact behavioral biases as they influence health decision-making including long-term ART adherence. Additionally, interventions can be designed combining different elements of material deprivations (e.g., food and income) as they can affect adherence-related behavior.

This study also has some limitations. Firstly, it does not explicitly establish the impact of deprivations on ART adherence since the reported outcomes are self-reported (and hypothetical), and therefore may suffer from self-reported bias. For example, people might report adherence levels under some scenarios that are different than how they would realistically adhere. Relatedly, the utility values estimated from the CA are not a validated measured of directly observed adherence since they are not necessarily about the behavior of preferences (or choices, as one would observe in a discrete choice experiment), and it is not clear how well they may predict clinical ART adherence. Secondly, while we can disentangle effects of the four poverty-related attributes, we cannot explain other channels through which they may influence adherence (e.g., food insecurity may act through other unmeasured factors). We are not showing whether these attributes cause behavioral biases or how these factors affect preferences and decision-making when it comes to ART adherence, but instead we explain which deprivations may affect probabilities of adherence. Thirdly, we cannot rule out participant measurement error resulting from respondent fatigue, social desirability bias, or misunderstanding of the attributes [37]. Participants may be reluctant to share which attributes affect their adherence, but we have no reason to think that this bias would vary differentially with respect to the different types of attributes under study. We made efforts to minimize such limitations by reducing time and cognitive load, i.e., we only included eight questions per block. We accounted for participant misunderstanding of attributes (or heterogeneous interpretation), and inattention (due to the hypothetical context of the study) by presenting some practice scenarios a priori and using attributes that many participants had previously listed as affecting their ART adherence. Lastly, we also recognize that measurement of income may be noisy due to the presence of formal and informal employment, and because respondents are disclosing personal income when they may not be the primary breadwinner (and this may not affect their adherence levels). Our study findings could be strengthened via a more robust measurement of income using assets (and possibly the creation of an asset index that can measure income or poverty levels more reliably at the household level).

The results from the CA can be valuable since they evaluate the relative influence of different levels of attributes on the same expected behavioral response (in terms of adherence). The findings imply that PLWH perceive external factors inherent in the lives of the poor and unrelated to ART access at the clinic to be important barriers to their expected ART adherence. Such attributes may also be valued in terms of adherence and can be meaningful to policymakers looking to mitigate barriers to ART adherence in different settings.

## Supporting information

S1 Checklist A checklist for conjoint analysis applications in health care. (DOCX)

S1 File CA protocol (enumerator instructions and example, and CA scenarios). (DOCX)

S2 File Main effects (covariate-adjusted). (DOCX)

S1 Questionnaire Inclusivity in global research. (DOCX)
