# Supplementary material for: The role of material deprivations in determining ART adherence: Evidence from a conjoint analysis among HIV-positive adults in Uganda
Source: PLOS Glob Public Health. 2022 Aug 17;2(8):e0000374. doi: 10.1371/journal.pgph.0000374 (PMC10022174; doi:10.1371/journal.pgph.0000374)
Supplement: S1 Checklist — (DOCX) [file pgph.0000374.s001.docx]

### S1 Checklist: A checklist for conjoint analysis applications in health care

Adapted from Bridges JFP, Hauber BA, Marshal D, Lloyd A, Prosser LA, Regier DA, et al. Conjoint Analysis Applications in Health – a Checklist: A Report of the ISPOR Good Research Practices for Conjoint Analysis Task Force. Value in Health 14 (2011): 403-413

1. Was a well-defined research question stated and is conjoint analysis an appropriate method for answering it?

1.1 Were a well-defined research question and a testable hypothesis articulated?

*Our research aimed to estimate the effects of various poverty-related factors such as material deprivations on ART adherence and disentangle the effects of these multi-faceted deprivations from one another*

1.2 Was the study perspective described, and was the study placed in a particular decision-making or policy context?

*We described that this study would be of interest to policymakers interested in designing interventions outside the clinic setting*

1.3 What is the rationale for using conjoint analysis to answer the research question?

*We aimed to be able to distinguish competing poverty-related factors that influence ART adherence. See Introduction*

2. Was the choice of attributes and levels supported by evidence?

2.1 Was attribute identification supported by evidence (literature reviews, focus groups, or other scientific methods)?

*We used a combination of qualitative data, baseline quantitative data, and literature review to identify attributes and their levels. Details are in the Design sub-section in Methods*

2.2 Was attribute selection justified and consistent with theory?

*As above*

2.3 Was level selection for each attribute justified by the evidence and consistent with the study perspective and hypothesis?

*As above*

3. Was the construction of tasks appropriate?

3.1 Was the number of attributes in each conjoint task justified (that is, full or partial profile)?

*We identified the attributes based on a combination of methods and designed the CA based on a fractional factorial design to avoid participant fatigue. This has been justified in literature as well: Street DJ, Burgess L, Louviere JJ. Quick and easy choice sets: Constructing optimal and nearly optimal stated choice experiments. International Journal of Research in Marketing. 2005;22(4):459-70*

3.2 Was the number of profiles in each conjoint task justified?

As above

3.3 Was (should) an opt-out or a status-quo alternative (be) included?

*Participants were free to refuse to answer for the scenario described, but an opt-out specific question was not included.*

4. Was the choice of experimental design justified and evaluated?

4.1 Was the choice of experimental design justified? Were alternative experimental designs considered?

*Please refer to Methods (Design)*

4.2 Were the properties of the experimental design evaluated?

*As above*

4.3 Was the number of conjoint tasks included in the data-collection instrument appropriate?

*We included 8 choices per person within each block, and randomized by block. This number seemed appropriate for statistical efficiency and response efficiency and to avoid participant fatigue.*

5. Were preferences elicited appropriately, given the research question?

5.1 Was there sufficient motivation and explanation of conjoint tasks?

*The protocol is described in S2.*

5.2 Was an appropriate elicitation format (that is, rating, ranking, or choice) used? Did (should) the elicitation format allow for indifference?

*As above*

5.3 In addition to preference elicitation, did the conjoint tasks include other qualifying questions (for example, strength of preference, confidence in response, and other methods)?

*No, they did not*

6. Was the data collection instrument designed appropriately?

6.1 Was appropriate respondent information collected (such as sociodemographic, attitudinal, health history or status, and treatment experience)?

*Yes, these data were available at baseline (almost 10-12 months prior to this CA and some additional data were obtained in the study survey conducted at the time of this CA)*

6.2 Were the attributes and levels defined, and was any contextual information provided?

*Please refer to Methods (Design)*

6.3 Was the level of burden of the data-collection instrument appropriate? Were respondents encouraged and motivated?

*See Protocol in S2*

7. Was the data-collection plan appropriate?

7.1 Was the sampling strategy justified (for example, sample size, stratification, and recruitment)?

*See Methods (Sample Size Determination and Study Setting and Recruitment)*

7.2 Was the mode of administration justified and appropriate (for example, face-to-face, pen-and-paper, web-based)?

*We administered the CA electronically to participants who were sitting close to coordinators for interviews conducted until February 2020. After that, we accounted for appropriate social distancing measures once study activities resumed in the wake of the COVID-19 pandemic. See Protocol for more details.*

7.3 Were ethical considerations addressed (for example, recruitment, information and/or consent, compensation)?

*Participants provided written consent for the study and accompanying surveys. The study received ethical approval from various IRBs (Methods section, Ethics Statement). None of the participants who were approached to do the CA refused that section or were unable to do so.*

8. Were statistical analyses and model estimations appropriate?

8.1 Were respondent characteristics examined and tested?

*Respondent characteristics are described in Table 1.*

8.2 Was the quality of the responses examined (for example, rationality, validity, reliability)?

*We did assess the quality of responses*

8.3 Was model estimation conducted appropriately? Were issues of clustering and subgroups handled appropriately?

*This is described in the Methods and Results sections*

9. Were the results and conclusions valid?

9.1 Did study results reflect testable hypotheses and account for statistical uncertainty?

*We include standard errors for all parameter estimates and include an additional regression specification as a robustness check*

9.2 Were study conclusions supported by the evidence and compared with existing findings in the literature?

*See Discussion*

9.3 Were study limitations and generalizability adequately discussed?

*See Discussion*

10. Was the study presentation clear, concise, and complete?

10.1 Was study importance and research context adequately motivated?

*See Introduction*

10.2 Were the study data-collection instrument and methods described?

*See Methods (Data Collection and Procedures)*

10.3 Were the study implications clearly stated and understandable to a wide audience?

*See Discussion*
