# Supplementary material for: The role of material deprivations in determining ART adherence: Evidence from a conjoint analysis among HIV-positive adults in Uganda
Source: PLOS Glob Public Health. 2022 Aug 17;2(8):e0000374. doi: 10.1371/journal.pgph.0000374 (PMC10022174; doi:10.1371/journal.pgph.0000374)
Supplement: S1 File — (DOCX) [file pgph.0000374.s002.docx]

### S1 CA Protocol: CA Protocol

#### Enumerator Instructions and Practice Example

Below is the general format for the Conjoint Analysis (CA), followed by a table with the different scenarios that will be randomized to each participant such that each participant is asked to choose what level of adherence s/he would anticipate under 8 different daily scenarios.

*Note: Red text is for coordinator to read out loud on screen*

**GENERAL FORMAT**

Screen 1:

Now for this section, I will walk you through a total of 8 imaginary scenarios with different characteristics and ask you the same question after each one. I will ask you to imagine a day like this. We can do some practice examples at first…..

(Instructions for coordinators:

You can clarify what each of the attributes mean, and if the participant says that one scenario looks the same as the one before it, you are allowed to point out why it is different

Do not ask or frame the question in a different way than the one explained

Do not suggest what respondents should answer)

Screen 2: “Now I will walk you through some imaginary scenarios:”

Screen 3: “Imagine a day when…

(Coordinator to read out scenarios described in box- scenarios are on following page)

“Are you able to imagine this type of day?”

(If participant says “No”, Coordinator reads out scenario again and clarify attributes)

Screen 4: Now, please rate your ability to take your HIV medications under these conditions on a day like this?

(Coordinator to select from options listed on screen).

(The six response options are “very poor,” “poor,” “fair,” “good,” “very good,” and “excellent.”)

Screens go through 7 more scenarios per participant, followed by question above after each section

Last Screen in this section: “Among these different conditions, which condition is the most important to in determining your adherence to your HIV medications?”

(The list will include: “Food Insecurity”, “Pain”, “Sleep”, “Money”)

**END OF GENERAL FORMAT**

**Example**

**Screen 1:**

“Now, I will walk you through some imaginary scenarios:”

**Screen 2: Scenario 1**

| Food Insecurity | Pain | Sleep | Money |
| --- | --- | --- | --- |
| Skipped 0 meals | No pain that makes it difficult to perform daily activities | 8 hours of sleep | Earned income of USh 200,000 in the previous month |

“Imagine a day when you have skipped 0 meals, you are in no physical pain, have had 8 hours of sleep last night, and you earned USh 200,000 in the previous month”

(pause and let the participant understand the situation)

“Are you able to imagine this type of day?”

**Screen 3: Scenario 1 response**

Now, please rate your ability to take your HIV medications under these conditions on a day like this?

Very Poor, Poor, Fair, Good, Very Good, Excellent

**Screen 4: Scenario 2**

| Skipped 0 meals | No pain that makes it difficult to perform daily activities | < 3 hours of sleep | Earned income of USh 100,000 in last month |
| --- | --- | --- | --- |

“Imagine a day when you have skipped 0 meals, you are in no physical pain, have had less than 3 hours of sleep last night, earned USh 100,000 in the last month”

(pause and let the participant understand the situation)

“Are you able to imagine this type of day?”

**Screen 5: Scenario 2 response**

Now, please rate your ability to take your HIV medications under these conditions on a day like this?

Very Poor, Poor, Fair, Good, Very Good, Excellent

**Screen 6: Scenario 3**

| Skipped 2 or more meals | No pain that makes it difficult to perform daily activities | 8 hours of sleep | No income in the last month |
| --- | --- | --- | --- |

“Imagine a day when you have skipped 2 or more meals, you are in no physical pain, have had regular sleep last night, had no income in the last month”

(pause and let the participant understand the situation)

“Are you able to imagine this type of day?”

**Screen 7: Scenario 3 response**

Now, please rate your ability to take your HIV medications under these conditions on a day like this?

Very Poor, Poor, Fair, Good, Very Good, Excellent

#### CA Scenarios

| Choice | Block | task | Food Insecurity | Pain | Sleep | Money |
| --- | --- | --- | --- | --- | --- | --- |
| 1 | 1 | 1 | Skipped 1 meal | Pain | Less than 2 hours of sleep | Monthly income of USh 100,000 |
| 2 | 1 | 2 | Skipped 2 or more meals | No pain | 3-5 hours of sleep | No monthly income |
| 3 | 1 | 3 | Skipped 2 or more meals | Pain | Regular sleep with no interruption | Monthly income of USh 100,000 |
| 4 | 1 | 4 | Skipped 0 meals | Pain | Regular sleep with no interruption | No monthly income |
| 5 | 1 | 5 | Skipped 2 or more meals | Pain | 3-5 hours of sleep | Monthly income of USh 100,000 |
| 6 | 1 | 6 | Skipped 0 meals | No pain | 3-5 hours of sleep | Monthly income of USh 200,000 |
| 7 | 1 | 7 | Skipped 0 meals | No pain | Less than 2 hours of sleep | No monthly income |
| 8 | 1 | 8 | Skipped 1 meal | No pain | Less than 2 hours of sleep | Monthly income of USh 200,000 |
| 9 | 2 | 1 | Skipped 0 meals | No pain | 3-5 hours of sleep | Monthly income of USh 100,000 |
| 10 | 2 | 2 | Skipped 1 meal | Pain | Less than 2 hours of sleep | No monthly income |
| 11 | 2 | 3 | Skipped 1 meal | No pain | 3-5 hours of sleep | Monthly income of USh 100,000 |
| 12 | 2 | 4 | Skipped 2 or more meals | Pain | 3-5 hours of sleep | No monthly income |
| 13 | 2 | 5 | Skipped 2 or more meals | Pain | Less than 2 hours of sleep | Monthly income of USh 100,000 |
| 14 | 2 | 6 | Skipped 0 meals | No pain | Less than 2 hours of sleep | No monthly income |
| 15 | 2 | 7 | Skipped 1 meal | Pain | Regular sleep with no interruption | Monthly income of USh 200,000 |
| 16 | 2 | 8 | Skipped 2 or more meals | No pain | Regular sleep with no interruption | Monthly income of USh 200,000 |
| 17 | 3 | 1 | Skipped 1 meal | No pain | 3-5 hours of sleep | Monthly income of USh 200,000 |
| 18 | 3 | 2 | Skipped 1 meal | Pain | Less than 2 hours of sleep | Monthly income of USh 200,000 |
| 19 | 3 | 3 | Skipped 0 meals | Pain | Regular sleep with no interruption | Monthly income of USh 200,000 |
| 20 | 3 | 4 | Skipped 2 or more meals | Pain | Less than 2 hours of sleep | No monthly income |
| 21 | 3 | 5 | Skipped 2 or more meals | No pain | Regular sleep with no interruption | No monthly income |
| 22 | 3 | 6 | Skipped 0 meals | No pain | Regular sleep with no interruption | No monthly income |
| 23 | 3 | 7 | Skipped 2 or more meals | No pain | Less than 2 hours of sleep | Monthly income of USh 100,000 |
| 24 | 3 | 8 | Skipped 0 meals | Pain | 3-5 hours of sleep | Monthly income of USh 100,000 |
| 25 | 4 | 1 | Skipped 0 meals | Pain | Less than 2 hours of sleep | Monthly income of USh 200,000 |
| 26 | 4 | 2 | Skipped 1 meal | Pain | Regular sleep with no interruption | No monthly income |
| 27 | 4 | 3 | Skipped 1 meal | No pain | 3-5 hours of sleep | No monthly income |
| 28 | 4 | 4 | Skipped 2 or more meals | No pain | 3-5 hours of sleep | Monthly income of USh 200,000 |
| 29 | 4 | 5 | Skipped 1 meal | No pain | Less than 2 hours of sleep | Monthly income of USh 200,000 |
| 30 | 4 | 6 | Skipped 0 meals | Pain | Regular sleep with no interruption | Monthly income of USh 100,000 |
| 31 | 4 | 7 | Skipped 2 or more meals | Pain | 3-5 hours of sleep | Monthly income of USh 100,000 |
| 32 | 4 | 8 | Skipped 2 or more meals | No pain | Less than 2 hours of sleep | No monthly income |
| 33 | 5 | 1 | Skipped 2 or more meals | Pain | 3-5 hours of sleep | No monthly income |
| 34 | 5 | 2 | Skipped 1 meal | No pain | Regular sleep with no interruption | Monthly income of USh 200,000 |
| 35 | 5 | 3 | Skipped 0 meals | Pain | 3-5 hours of sleep | Monthly income of USh 200,000 |
| 36 | 5 | 4 | Skipped 1 meal | Pain | Regular sleep with no interruption | Monthly income of USh 100,000 |
| 37 | 5 | 5 | Skipped 1 meal | No pain | Less than 2 hours of sleep | Monthly income of USh 100,000 |
| 38 | 5 | 6 | Skipped 0 meals | No pain | 3-5 hours of sleep | Monthly income of USh 100,000 |
| 39 | 5 | 7 | Skipped 2 or more meals | Pain | Regular sleep with no interruption | Monthly income of USh 200,000 |
| 40 | 5 | 8 | Skipped 0 meals | Pain | Less than 2 hours of sleep | No monthly income |
| 41 | 6 | 1 | Skipped 0 meals | Pain | Regular sleep with no interruption | Monthly income of USh 200,000 |
| 42 | 6 | 2 | Skipped 2 or more meals | No pain | Regular sleep with no interruption | Monthly income of USh 100,000 |
| 43 | 6 | 3 | Skipped 1 meal | Pain | 3-5 hours of sleep | No monthly income |
| 44 | 6 | 4 | Skipped 2 or more meals | No pain | Less than 2 hours of sleep | No monthly income |
| 45 | 6 | 5 | Skipped 0 meals | Pain | Less than 2 hours of sleep | Monthly income of USh 200,000 |
| 46 | 6 | 6 | Skipped 1 meal | Pain | 3-5 hours of sleep | No monthly income |
| 47 | 6 | 7 | Skipped 2 or more meals | No pain | Less than 2 hours of sleep | Monthly income of USh 200,000 |
| 48 | 6 | 8 | Skipped 1 meal | No pain | Regular sleep with no interruption | Monthly income of USh 100,000 |
| 49 | 7 | 1 | Skipped 1 meal | Pain | Regular sleep with no interruption | Monthly income of USh 100,000 |
| 50 | 7 | 2 | Skipped 1 meal | No pain | 3-5 hours of sleep | Monthly income of USh 100,000 |
| 51 | 7 | 3 | Skipped 2 or more meals | Pain | Regular sleep with no interruption | Monthly income of USh 200,000 |
| 52 | 7 | 4 | Skipped 0 meals | No pain | Regular sleep with no interruption | No monthly income |
| 53 | 7 | 5 | Skipped 0 meals | No pain | 3-5 hours of sleep | Monthly income of USh 200,000 |
| 54 | 7 | 6 | Skipped 1 meal | Pain | Less than 2 hours of sleep | No monthly income |
| 55 | 7 | 7 | Skipped 0 meals | No pain | Less than 2 hours of sleep | Monthly income of USh 100,000 |
| 56 | 7 | 8 | Skipped 2 or more meals | Pain | 3-5 hours of sleep | Monthly income of USh 200,000 |
| 57 | 8 | 1 | Skipped 1 meal | Pain | 3-5 hours of sleep | Monthly income of USh 200,000 |
| 58 | 8 | 2 | Skipped 2 or more meals | Pain | Less than 2 hours of sleep | Monthly income of USh 100,000 |
| 59 | 8 | 3 | Skipped 0 meals | Pain | 3-5 hours of sleep | No monthly income |
| 60 | 8 | 4 | Skipped 2 or more meals | No pain | Less than 2 hours of sleep | Monthly income of USh 200,000 |
| 61 | 8 | 5 | Skipped 2 or more meals | No pain | Regular sleep with no interruption | No monthly income |
| 62 | 8 | 6 | Skipped 0 meals | Pain | Less than 2 hours of sleep | Monthly income of USh 100,000 |
| 63 | 8 | 7 | Skipped 1 meal | No pain | Regular sleep with no interruption | No monthly income |
| 64 | 8 | 8 | Skipped 0 meals | No pain | Regular sleep with no interruption | Monthly income of USh 100,000 |
