# Supplementary material for: The role of material deprivations in determining ART adherence: Evidence from a conjoint analysis among HIV-positive adults in Uganda
Source: PLOS Glob Public Health. 2022 Aug 17;2(8):e0000374. doi: 10.1371/journal.pgph.0000374 (PMC10022174; doi:10.1371/journal.pgph.0000374)
Supplement: S2 File — (DOCX) [file pgph.0000374.s003.docx]

### S1 Main Effects: Main Effects (covariate-adjusted)

| **Attribute** | **Level** | **1** | | |
| --- | --- | --- | --- | --- |
|  |  | **Coefficient** | **95% CI** | **p-value** |
| Food Insecurity | Skipped 0 meals | 1.308 | 1.124 - 1.492 | <0.001 |
|  | Skipped 1 meal | 0.878 | 0.740 - 1.015 | <0.001 |
| Sleep | 3-5 hours of sleep | 0.101 | 0.00785 - 0.194 | <0.05 |
|  | 8 hours of sleep | 0.338 | 0.208 - 0.467 | <0.001 |
| Income | Earned income of Ush 100,000 in last month | 0.808 | 0.718 - 0.897 | <0.001 |
|  | Earned income of Ush 200,000 in last month | 0.989 | 0.879 - 1.099 | <0.001 |
| Pain | No pain that makes it difficult to perform daily activities | 0.391 | 0.274 - 0.508 | <0.001 |
| R-squared |  | 0.2697 |  |  |
| Observations |  | 2,544 |  |  |
| Number of participants |  | 320 |  |  |
| Number of blocks |  | 8 |  |  |
| Note: Column 1 shows covariate-adjusted results from the CA sample analyzed using an OLS regression specification. All coefficients represent improvements in expected ART adherence (coefficients in ordered logit column represent odds ratios) and are compared to the reference categories (Food Insecurity: Skipped 2 meals, Sleep: 0 hours of sleep; Income: No income; Pain: pain that makes it difficult to perform daily activities). Covariates include participant sex (male or female), age, education (secondary or more), employment status (binary variable), depressed status, intrinsic motivation scale, food insecure, years at Mildmay, years taking ART, whether participant felt comfortable disclosing HIV status to friends or family, and whether the participant was impatient or present-biased (variables are described in more detail in Panel B, Table 2). 95% confidence intervals and p-values are shown adjacent to coefficients. Standard errors were clustered at the individual level. | | | | |
